# Reproducibility of carotid ultrasound measurements in the Brazilian Longitudinal Study of Adult Health (ELSA-Brasil) at baseline

**DOI:** 10.1590/1414-431X20198711

**Published:** 2019-08-05

**Authors:** P.J. Santos-Neto, E.H. Sena-Santos, D.P. Meireles, I.S. Santos, I.M. Bensenor, P.A. Lotufo

**Affiliations:** 1 Centro de Pesquisa Clínica e Epidemiológica, Hospital Universitário, Universidade de São Paulo, São Paulo, SP, Brasil; 2 Departamento de Clínica Médica, Faculdade de Medicina, Universidade de São Paulo, São Paulo, SP, Brasil

**Keywords:** Plaque, Intima-media, Epidemiology, Ultrasound, Imaging

## Abstract

Carotid artery assessment by ultrasound is a non-invasive evaluation of subclinical atherosclerosis and a predictor of cardiovascular events. However, ultrasound examinations are operator-dependent. In the Brazilian Longitudinal Study of Adult Health (ELSA-Brasil), ultrasound images have been acquired from more than 10,000 participants. In this article, we describe the reproducibility of carotid intima-media thickness (CIMT), carotid plaque detection, and carotid plaque score (defined as the number of arterial sites with plaques) using ELSA-Brasil protocol, in a subset of 118 participants. Two board-certified radiologists and a trained technician read carotid images. We calculated intra- and inter-observer intraclass correlation (ICC) for CIMT values. We also present kappa coefficients for plaque detection and weighted kappa coefficients for carotid plaque score. Participants were aged 58.2±6.6 years, and 60 (50.8%) were men. For common carotid artery CIMT measurements, intra- and inter-observer ICC values were very good to excellent, ranging from 0.90 (95% confidence interval [95%CI]: 0.72–0.95) to 0.98 (95%CI: 0.97–0.99). For carotid plaque, intra- (0.96 [95%CI: 0.96–0.96]) and inter- (0.99 [95%CI: 0.99–0.99]) observer weighted kappa coefficients were very good. Intra- and inter-observer Kappa coefficients for the presence of plaques by site were good to very good, ranging from 0.69 to 1.00. In conclusion, we found very good reproducibility for carotid plaque score and CIMT measurements in the ELSA-Brasil at baseline. These results are comparable to the best findings from similar large cohorts that analyzed carotid ultrasound data.

## Introduction

Ultrasonographic evaluation of carotid artery plaques and carotid intima-media thickness (CIMT) are increasingly used to assess the extent and severity of subclinical atherosclerosis (1–3). The ultrasound exam is widely-available, safe, and noninvasive; however, its reproducibility depends on machine and operator factors (1,2). B-mode image quality varies with interrogation angle, machine capabilities, and fine adjustment of instrument controls (1,2). Disagreement among readers regarding the transition line from common carotid to carotid bulb may lead to divergent CIMT measurements and disagreement as to the location of plaques (1).

In the Brazilian Longitudinal Study of Adult Health (ELSA-Brasil) (4–7) study, ultrasound images have been acquired for more than 10,000 participants at baseline, with strict protocols for image acquisition and reading (4).The aim of this article is to describe the intra- and inter-observer reproducibility of CIMT, carotid plaque detection, and carotid plaque score, using ELSA-Brasil protocol for ultrasound imaging acquisition and reading.

## Material and Methods

ELSA-Brasil is a prospective cohort of 15,105 civil servants from 6 different Brazilian cities (São Paulo, Belo Horizonte, Rio de Janeiro, Porto Alegre, Salvador, and Vitoria), aged 35 to 74 years. It was designed to evaluate cardiovascular diseases and diabetes, as well as their biological and social determinants (4–7). The baseline assessments occurred from August 2008 to December 2010. Approvals of the ethics committees were granted, and all individuals provided their written informed consent (4,5).

All participants were examined by trained staff (4,5). The images were obtained by a Toshiba Aplio XG ultrasound device (Japan) with a linear 7.5 MHz transducer, between August 2008 and December 2010 (4,5). Images were taken of the longitudinal axis of the common carotid artery (CCA), bulb, and origin of the internal carotid artery (ICA), observing three electrocardiographically gated cardiac cycles. Images were also obtained of the short (transverse) axis of all visible segments, including the bulb and ICA, up to the mandibular branch. All the images obtained were sent to an examination reading center in São Paulo (4,5). The definitions of CIMT and plaques assumed by the ELSA-Brasil protocol (4,5) are those adopted by the Mannheim consensus (2) and the Echocardiography American Society (1).

### Measurement of CIMT

To measure CIMT, each CCA was identified along its longitudinal axis, using standard brightness and contrast. CIMT was semi-automatically calculated using Medical Imaging Applications software (MIA-LLC, USA), analyzing three electrocardiographically gated cardiac cycles (4,5). Only the far wall of the CCA (1 cm proximal to the carotid bulb, with 1 cm in length) was measured, and minimum, mean, and maximum values for each CCA were obtained (4,5). The current Brazilian recommendations for ultrasound evaluation of carotid atherosclerotic disease adopts the ELSA-Brasil CIMT values as standard for the Brazilian adult population (8).

### Measurement of plaque

The near and far walls of the CCA, carotid bulb, and origin of the ICA bilaterally were evaluated longitudinally and transversally for plaque detection, following the Mannheim consensus criteria (2). Plaques were evaluated by visual judgment, without measuring the thickness of the lesions (9–14). All 12 combinations of side (left, right), wall (near, far), and segment (CCA, bulb, ICA) were evaluated for the presence or absence of plaques. The count of sites that presented plaques yielded a score ranging from 0 to 12 (13,14).

### Study sample

A random subsample of 118 participants from the ELSA-Brasil São Paulo site with valid left and right carotid ultrasound images was selected for this study (4,6,7). The size of this subsample was similar to that of other reproducibility studies (9,11,13,14). The same certified technician (DPM) assessed image quality (15,16) for all acquisitions.

For the purpose of the present study, readings were performed offline between October 2016 and March 2017, at the reading center in São Paulo. Two board-certified radiologists (PJSN, EHSS) read the plaque images using the same protocol. One radiologist (PJSN) and a trained technician with more than 5 years of experience (DPM) read the CIMT images. Readings for intra-observer agreement were performed with a minimum interval of 3 months between the first and second readings. All staff were blinded to the participants' clinical information.

### Statistical analysis

We calculated intraclass correlation (ICC) for CIMT to estimate intra- and inter-observer agreement (15–17). ICC estimates were based on single-rating, absolute-agreement, two-way models. We verified residual distribution and potentially influential measurements using DFFITS, residual Q-Q plots, residual x-fitted plots, and Shapiro-Wilk normality tests. ICC coefficients were slightly higher when using datasets after the exclusion of influential points, compared to the use of the entire dataset. To use the same set of observations throughout the paper (enhancing comparability among measurements) and to report conservative results, avoiding bias toward the exclusion of observations with greater disagreement, we opted to report the results from models using the entire dataset as our main results.

We also report kappa coefficients (17) for intra- and inter-observer agreement in the presence of plaque in each of the twelve possible sites in the carotid arteries and weighted kappa coefficients for intra- and inter-observer agreement in plaque score. ICC values <0.50, between 0.50 and 0.75, between 0.76 and 0.90, and >0.90 are indicative of poor, moderate, good, and excellent reliability, respectively (15). Kappa coefficients ≤0.20, between 0.21 and 0.40, between 0.41 and 0.60, between 0.61 and 0.80, and >0.80 are classified as poor, fair, moderate, good, and very good reliability, respectively (17). We report 95% confidence intervals (95%CI). Whenever the 95%CI for kappa coefficients reached 1.0, this result was confirmed by bootstrapping. All analyses were performed using R software, boot, irr, and psych packages.

## Results

The studied sample was aged 58.2±6.6 years, 60 (50.8%) were men, 57 (48.3%) had hypertension, 32 (27.1%) had diabetes, 22 (18.6%) were current smokers, and the mean body mass index was 27.3±4.4 kg/m^2^. Table 1 shows the intra- and inter-observer agreement for CIMT and plaque measurements. The mean CIMT and plaque scores had excellent intra- and inter-observer agreement. Maximum and minimum CIMT had very good to excellent intra- and inter-observer agreement. Kappa coefficients for the presence of plaques by site ranged from 0.69 to 1.00 (good to very good).

**Table 1:** Intraclass correlation and kappa coefficients (95%CI) for intra- and inter-observer agreement in carotid intima-media thickness (CIMT) and plaque measurements.

CIMT measurements	Intraclass correlation coefficients (95%CI)	
	Intra-observer	Inter-observer
Left CCA, minimum	0.93 (0.89–0.95)	0.91 (0.81–0.95)
Left CCA, mean	0.98 (0.97–0.98)	0.98 (0.97–0.99)
Left CCA, maximum	0.95 (0.93–0.97)	0.94 (0.73–0.98)
Right CCA, minimum	0.92 (0.88–0.95)	0.93 (0.84–0.97)
Right CCA, mean	0.97 (0.96–0.98)	0.98 (0.97–0.98)
Right CCA, maximum	0.96 (0.95–0.97)	0.90 (0.72–0.95)

Plaque measurements	Kappa coefficients (95%CI)	
	Intra-observer	Inter-observer
Plaque score	0.96 (0.96–0.96)	0.99 (0.99–0.99)
Presence of plaque by site		
Left CCA, near wall	0.87 (0.72–1.00)	0.95 (0.86–1.00)
Left CCA, far wall	0.77 (0.60–0.93)	0.93 (0.83–1.00)
Left carotid bulb, near wall	0.90 (0.81–0.98)	0.84 (0.73–0.95)
Left carotid bulb, far wall	0.81 (0.71–0.92)	0.93 (0.86–1.00)
Left ICA, near wall	0.84 (0.67–1.00)	0.90 (0.76–1.00)
Left ICA, far wall	0.79 (0.65–0.94)	0.97 (0.91–1.00)
Right CCA, near wall	0.76 (0.49–1.00)	1.00 (1.00–1.00)
Right CCA, far wall	0.76 (0.53–0.99)	0.94 (0.81–1.00)
Right carotid bulb, near wall	0.83 (0.69–0.96)	0.92 (0.83–1.00)
Right carotid bulb, far wall	0.81 (0.69–0.92)	0.98 (0.94–1.00)
Right ICA, near wall	0.85 (0.64–1.00)	0.76 (0.53–0.99)
Right ICA, far wall	0.69 (0.47–0.90)	0.81 (0.64–0.99)

CCA: common carotid artery; ICA: internal carotid artery.

## Discussion

The present analyses show excellent intra- and inter-observer reproducibility for the carotid plaque score and mean CIMT measurements in the ELSA-Brasil. Mean CIMT and plaque scores were more reliable measurements in this study than maximum and minimum CIMT, perhaps because the former reduces the impact of extreme values.

CIMT measurement is challenging, and protocols among studies vary. Far wall measurements are usually preferred because their accuracy has been validated against CCA histological specimens (18). The phase of the cardiac cycle also differs, and influences the values obtained. Due to expansion of the systolic lumen diameter, which leads to thinning of CIMT during systole, CIMT values obtained from end-systole are lower than those obtained in end-diastole (19). Another factor to be considered is that computed assisted CIMT measurements are more accurate than manual evaluations (20). All these considerations were used to design the ELSA-Brasil protocol, with a semi-automated CIMT measurement in the far CCA wall, analyzing three electrocardiographically gated cardiac cycles (4). It is likely that these factors contributed to the good outcomes of the ELSA-Brasil on CIMT reproducibility.

There is also a lack of agreement on the best way to analyze carotid plaques (1–3). Plaque thickness, score, area, or volume (by 3-dimensional ultrasound imaging) may be more accurate in predicting cardiovascular risk than the presence of plaques (3,9,11–14). However, plaque score analyses are practical because visual judgement alone is sufficient (9,11,13,14). When analyzing individual arterial sites for the presence of carotid plaques, we found some degree of intra- and inter-observer disagreement, albeit small in most cases. However, the ICC for the plaque score was excellent (0.959 and 0.961 for intra- and inter-observer agreement, respectively). This is mainly because, for some plaques, radiologists are unequivocal about their presence, but they may disagree about their exact segment location. This reinforces the plaque score as a reliable measurement of plaque burden, compared to the analysis of individual sites.

Other large cohorts, like the ARIC (Atherosclerosis Risk in Communities cohort) (10,12) and MESA (Multi-ethnic Study of Atherosclerosis cohort) (13,14) published their reproducibility results for plaque data and CIMT. They assessed the presence or absence of plaques in each segment of the carotid arteries (CCA, bulb, and ICA) on both sides, as well as measured CIMT in the far CCA wall. Our results were in agreement with the outcomes presented in that work.

Li et al. (10) analyzed carotid images from the ARIC, a population-based cohort of subjects between 45 and 64 years of age from four U.S. communities with baseline assessment between 1987 and 1989. The authors found ICC for a mean CIMT of 0.70 (10,12). For the presence of plaques, kappa coefficients for intra- and inter-observer agreement were 0.76 and 0.56, respectively (10,12). Possible explanations for these coefficients are associated with limitations in the equipment and software available at the time of the ARIC image acquisition and reading (10,12). Since the early 1990s, there have been important improvements in ultrasound technology, resulting in high-quality images and improved readings.

Tattersall et al. (14) analyzed carotid images of participants from the MESA study (14). MESA enrolled individuals aged 45–84 years, from different ethnic backgrounds (White, African-American, Hispanic, and Chinese-American), from 6 U.S. field centers, with baseline assessment between 2000 and 2002 (13,14). Analyzing the mean CIMT with semi-automated border detection software, (14) the authors showed that the ICC values for intra- and inter-observer agreement were 0.99 and 0.95, respectively (14). For carotid plaque presence and score, overall kappa coefficients for intra- and inter-observer agreement were 0.83 and 0.89, respectively (14). These results are very close to ours, suggesting a reliable methodology in both cohorts.

In conclusion, we found very good reproducibility for carotid plaque score and CIMT measurements in the ELSA-Brasil study. These results are comparable to the best findings from similar large cohorts that analyzed carotid ultrasound data.
